# Bee Venom Phospholipase A2 Induces Regulatory T Cell Populations by Suppressing Apoptotic Signaling Pathway

**DOI:** 10.3390/toxins12030198

**Published:** 2020-03-22

**Authors:** Hyunjung Baek, Seon-Young Park, Su Jeong Ku, Kihyun Ryu, Younsub Kim, Hyunsu Bae, Ye-Seul Lee

**Affiliations:** 1 Department of Physiology, College of Korean Medicine, Kyung Hee University, Seoul 02447, Korea; bguswjd@khu.ac.kr (H.B.); psys12@naver.com (S.-Y.P.); mediolateral@naver.com (K.R.); 2 Department of Anatomy and Acupoint, College of Korean Medicine, Gachon University, Seongnam 13120, Korea; revcandy@daum.net (S.J.K.); ysk@gachon.ac.kr (Y.K.)

**Keywords:** bee venom phospholipase A2, bvPLA2, regulatory T cells, Tregs, apoptosis

## Abstract

Bee venom phospholipase A2 is a lipolytic enzyme in bee venom that catalyzes hydrolysis of the sn-2 ester bond of membrane phospholipids to produce free fatty acid and lysophospholipids. Current evidence suggests that bee venom phospholipase A2 (bvPLA2) induces regulatory T cell expansion and attenuates several immune system-related diseases, including Alzheimer’s disease. The induction of Treg cells is directly mediated by binding to mannose receptors on dendritic cells. This interaction induces the PGE2-EP2 signaling pathway, which promotes Treg induction in CD4^+^ T cells. In this study, we investigated the effects of bvPLA2 treatment on the apoptotic signaling pathway in Treg populations. Flow cytometry was performed to identify early apoptotic cells. As a result, early apoptotic cells were dramatically decreased in bvPLA2-treated splenocytes, whereas rapamycin-treated cells showed levels of apoptotic cells similar to those of PBS-treated cells. Furthermore, bvPLA2 treatment increased expression of anti-apoptotic molecules including CTLA-4 and PD-1. The survival rate increased in bvPLA2-treated Tregs. Our findings indicate that bvPLA2-mediated modulation of apoptotic signaling is strongly associated with the Treg induction, which exhibits protective effects against various immune-related diseases. To our knowledge, this study is the first to demonstrate that bvPLA2 is the major bee venom (BV) compound capable of inducing Treg expansion through altering apoptotic signal.

## 1. Introduction

Bee venom (BV) is a complex substance extracted from the honeybee (*Apis mellifera* L.) which contains a variety of enzymes, biologically active amines, peptides, and non-peptide components. The two major compounds in BV are melittin and phospholipase A2 (bvPLA2). Melittin is a cationic cell-lytic peptide consisting of 26 amino acids, and bvPLA2 is an enzyme which hydrolyzes membrane phospholipids. Bee venom therapy (BVT) consists in using BV for therapeutic purposes via injection by either stings from live bees or acupuncture needles. BVT has recently been widely used as an alternative therapy for the clinical treatment of a number of diseases, including rheumatoid arthritis and Parkinson’s disease (PD) [1,2]. The anti-apoptosis, anti-fibrosis, and anti-inflammatory effects of BVT have been known for centuries and demonstrated in several reports and research articles [3,4,5]. However, adverse effects of BVT including skin problems, systemic reactions, and nonspecific reactions have also been reported. Furthermore, the mechanisms underlying its therapeutic effect are yet to be elucidated.

The enzyme bvPLA2 is a disulfide-rich enzyme that catalyzes the hydrolysis of the sn-2 ester bond of phospholipids, which leads to the release of arachidonic acid and lysophospholipids; bvPLA2 is part of the group III secretory PLA2 (sPLA2), and its catalytic activity is necessary to initiate the IgE response in rodent mast cells [6]. Moreover, bvPLA2 is a potent inducer of T helper type 2 (Th2) immune reactions and the activation of group 2 innate lymphoid cells (ILC2) through the release of IL-35 [7]. Recently, numerous studies have reported the protective effects of bvPLA2 against a variety of diseases, including Alzheimer’s disease, allergic asthma, and cisplatin-induced organ inflammation [8,9,10]. Chung et al. revealed that bvPLA2 binds to a mannose receptor (CD206) or a C-type lectin on dendritic cells (DC) and increases the expression of prostaglandin E2 (PGE2), which binds to EP2 receptors on naïve T helper cells and leads to the differentiation into CD4^+^CD25^+^Foxp3^+^ regulatory T cells (Tregs) [11]. The induction of Treg populations by bvPLA2 treatment contributes to immune suppression in various immune disease-related models [8,12].

Tregs are a specialized population of T cells for which the essential role is to maintain immune homeostasis and self-tolerance. Tregs are divided into three different types depending on the expression level of CD62L and CD127: resting Tregs (CD62L^hi^CD127^low^), activated effector Tregs (CD62L^low^CD127^low^), and memory Tregs (CD62L^low^CD127^hi^) [13]. Tregs have been shown to play a crucial role in the normal immune system for the establishment and maintenance of immunologic self-tolerance and immune homeostasis [14,15,16]. Adoptive transfer of ex vivo expanded CD4^+^CD25^+^ Tregs prevents pathology in various mouse models including graft versus host disease (GvHD) [17,18], and GvHD was the first disease to be tried in translation studies in humans. The clinical trial actually involved mainly stem cell transplantation (SCT), solid organ transplantation, and autoimmune diseases [19]. While previous studies implicated Tregs as an important constituent of the immune system for the maintenance of self-tolerance [14,15,16,20], a recent study further indicated that systemic administration of Tregs attenuates the progression of Alzheimer’s disease (AD) and could be an effective treatment [21]. In light of this finding, modulation of the Treg cells expansion has been proposed as a potential treatment for neuroinflammation-mediated diseases such as Parkinson’s disease and amyotrophic lateral sclerosis [22,23].

For the development of Tregs therapy for the treatment of autoimmune diseases and GvHD, the survival rate of Tregs in human body and the proliferation of Foxp3 Tregs in vivo are the important issues [24]. Tregs expanded ex vivo from allogeneic or autologous donors under good manufacturing practice (GMP) conditions retain antigen specificity, immune-suppressive properties, and favorable homing markers. However, ex vivo expanded human Tregs are heterogenic populations and comprise several subpopulations [25]. Recent studies reported the possibility of Foxp3^+^ Tregs losing its Foxp3 expression in vivo from epigenetic modifications of Foxp3, which generates inflammatory cytokine-producing pathogenic memory T cells [26]. Therefore, more homogenous and safer Treg-cell expansion protocols are required for successful Treg cell therapy.

The purpose of this study was to explore the potential mechanism underlying bvPLA2-mediated Treg induction. In our study, Annexin V^+^ and caspase-3^+^ early apoptotic cells were dramatically decreased in bvPLA2-treated splenocytes, whereas rapamycin-treated cells showed levels of apoptotic cells similar to those of PBS-treated cells. Furthermore, bvPLA2 treatment upregulated anti-apoptotic molecules such as PD-1 and CTLA-4. The anti-apoptotic effects of bvPLA2-treated Tregs in vivo was proven through the increased survival rates of Tregs in the adoptive transferred Treg population in vivo. This finding indicates that bvPLA2-mediated modulation of apoptotic signaling is strongly associated with the Treg induction, which exhibits protective effects against various immune-related diseases.

## 2. Results

### 2.1. bvPLA2-Mediated Induction of CD4^+^CD25^+^Foxp3^+^ Treg Cells in Vitro

**Figure 1:**
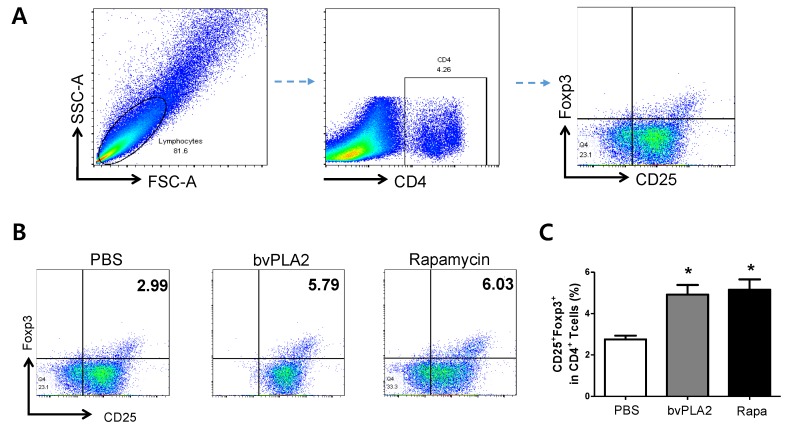
Effect of bee venom phospholipase A2 (bvPLA2) treatment on CD4^+^CD25^+^Foxp3^+^ Treg populations in in vitro splenocyte cultures. Single cell suspension of splenocytes were isolated from Foxp3EGFPC57BL/6 mice and stimulated with PBS, bvPLA2, and rapamycin in the presence of anti-CD3/CD28 antibodies. (**A**) The gating strategy was depicted. (**B**) After 72 h, cells were labeled with anti-CD4-PE-Cy7 and anti-CD25-APC-Cy7, and analyzed by flow cytometry. Lymphocytes were initially gated by forward and side scatter properties. CD4^+^ were then gated and assessed for surface expression of CD25 and FoxP3. (**C**) The percentages of CD4^+^CD25^+^Foxp3^+^ Tregs were depicted as the bar graph. The data are shown as the means ± SEM. Rapa, rapamycin. The significance was determined by one-way ANOVA followed by Tukey’s multiple comparison test. *p < 0.05 vs. the PBS group.

Previous reports showed that treatment with bvPLA2 induce the expansion of immunosuppressive Tregs in vitro and in vivo [8]. Flow cytometry was conducted to investigate the effect of bvPLA2 treatment on the induction of Tregs in vitro. To identify Tregs, CD4^+^ lymphocytes were gated from a total cell population, and CD25^+^ and Foxp3^+^ populations were further gated. As shown in Figure 1, the percentage of CD4^+^CD25^+^Foxp3^+^ Treg populations was significantly higher in bvPLA2 and rapamycin-treated groups compared with those in the PBS-treated group. No significant differences between bvPLA2 and rapamycin treatment were seen in the splenocyte culture.

### 2.2. bvPLA2-Mediated Phenotypical Changes of CD4^+^CD25^+^Foxp3^+^ Treg Cells

**Figure 2:**
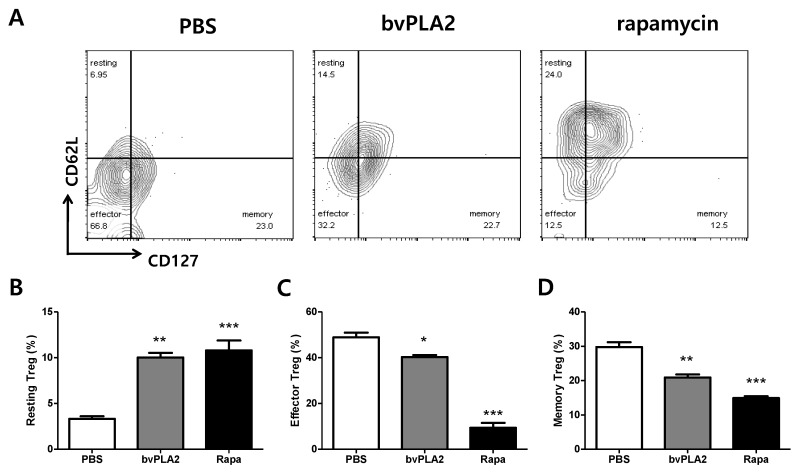
Effects of bvPLA2 treatment on Treg subpopulations in in vitro splenocyte cultures. (**A**) Splenocytes were stained with Treg cell phenotyping markers (CD4, CD25, Foxp3, CD62L, and CD127) and analyzed for Treg cell subsets by flow cytometry. Comparison of (**B**) resting Treg cell subsets (CD62LhiCD127low), (**C**) activated effector Treg cell subsets (CD62LlowCD127low), and (**D**) memory Treg cell subsets (CD62LlowCD127hi) based on expressions of CD62L and CD127 in PBS, bvPLA2, and rapamycin-treated splenocytes. Rapa, rapamycin. The data are shown as the means ± SEM. The significance was determined by one-way ANOVA followed by Tukey’s multiple comparison test. * *p* < 0.05, ** *p* < 0.01, and *** *p* < 0.001 vs. the PBS group.

Markers that classify resting, effector, and memory Treg cells have been identified. We analyzed three subsets of CD4^+^CD25^+^Foxp3^+^ Treg cells based on the expression of CD62L (L-selectin) and CD127. The results showed that the percentage of resting Treg cells was higher in bvPLA2 and rapamycin-treated cultures than in the PBS group (Figure 2A). Activated effector and memory Treg subpopulations instead declined in bvPLA2-treated splenocytes compared with the control (Figure 2B–D). Interestingly, the changes in the three Treg cell subsets showed similar patterns in both bvPLA2 and rapamycin-treated groups.

### 2.3. bvPLA2-Mediated Induction of Treg Cells Correlates with Apoptosis

**Figure 3:**
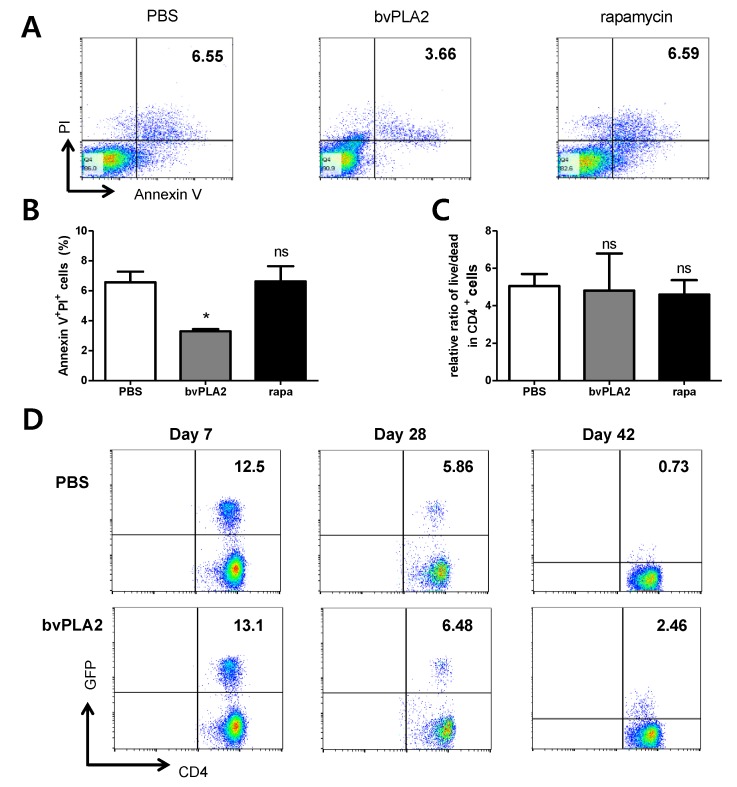
Apoptosis of Treg cells was negatively correlated with bvPLA2 treatment and Treg induction. Splenocytes isolated from Foxp3EGFPC57BL/6 mice were stimulated with plate bound anti-CD3 (5 μg/mL) and anti-CD28 (2 μg/mL) antibodies plus PBS, bvPLA2, or rapamycin for 72 h. Apoptosis analysis of PBS, bvPLA2, and rapamycin-treated splenocytes detected based on Annexin V and propidium iodide (PI) staining. (**A**) The expression of PI and Annexin V were assessed in CD4^+^ T cells. (**B**) The percentages of Annexin V^+^PI^+^ cells were depicted as the bar graph. (**C**) No correlation was observed in the relative ratio of live (Annexin V^−^PI^−^)/dead (PI^+^) CD4^+^ T cells. (**D**) 1 × 10^6^ FoxP3-GFP^+^ Tregs were adoptively transferred to C57BL/6 mice. After 7, 28, and 42 days, GFP^+^ adoptively transferred Tregs were detected in splenocytes. Representative dot-plots showed the expression of GFP in CD4^+^ T cells. Rapa, rapamycin. The data are shown as the means ± SEM. The significance was determined by one-way ANOVA followed by Tukey’s multiple comparison test. * *p* < 0.05 vs. the PBS group.

An important and delicate balance exists between Treg homeostasis and apoptosis in CD4^+^ T cells. Therefore, we further investigated whether bvPLA2 promotes T cell death in vitro by combining Annexin V with propidium iodide (PI) staining. Annexin V binds to apoptotic cells and PI stains late-stage apoptotic cells and necrotic cells. Splenocytes were Annexin V- and PI-positive in both PBS and rapamycin-treated cultures (Figure 3A,C). However, the overall number of Annexin and PI double-positive cells was significantly lower in bvPLA2-treated cultures (Figure 3B). A negative correlation was found between bvPLA2 treatment and the absolute count as well as the percentage of the apoptotic Treg cells. No correlation was observed between apoptotic Treg cell frequency and rapamycin treatment. Subsequently, in order to confirm the anti-apoptotic effects of bvPLA2-treated Tregs in vivo, the survival rates of Tregs were assessed by examining the adoptive transferred Treg population in vivo. The results showed that bvPLA2-treated Tregs survived for a longer period than the untreated Tregs in mice (Figure 3D).

### 2.4. Effect of bvPLA2 Treatment on Early Apoptosis in Splenocytes

**Figure 4:**
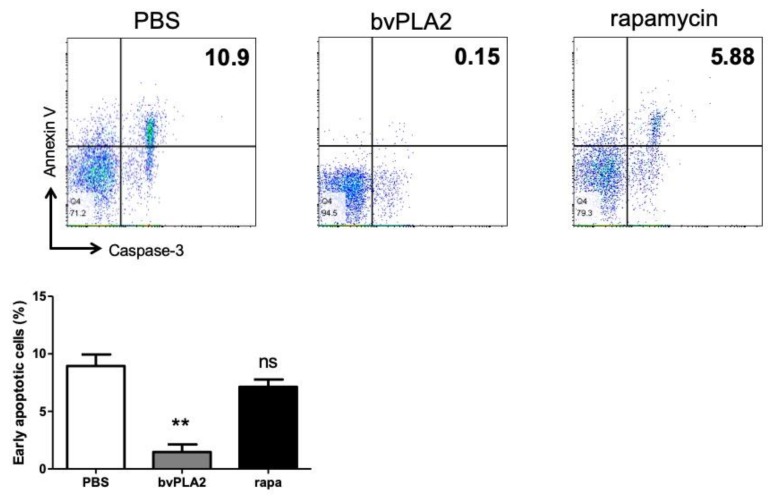
Effect of bvPLA2 on early apoptosis in splenocytes. Single-cell suspension splenocytes were treated with bvPLA2 or rapamycin for 72 h, stained with Annexin-V, 7-AAD, and active-caspase-3 antibody, and analyzed using flow cytometry. Live cells were gated as 7-AAD negative populations. The early apoptotic cells were depicted as Annexin V and active-caspase-3 double-positive populations.

Caspase activation is an important step in the onset of apoptosis. A recently described method for early apoptosis detection consists in using the novel cell-permeable fluorogenic caspase-3/7 substrate, which is a four-amino acid peptide (DEVD) conjugated to a nucleic acid-binding dye. Here, we used the fluorogenic caspase-3/7 substrate together with APC-conjugated Annexin V and the DNA binding dye 7-AAD to measure cell death. The resulting early apoptotic subpopulations were measured as caspase-3/7 and Annexin V double-positive populations after 7-AAD-negative gating. When cells were gated for negative labeling with 7-AAD, about 10% of caspase-3/7 and Annexin V double-positive populations appeared in the PBS group (Figure 4). Early apoptotic cells also appeared in the rapamycin group. However, a dramatic reduction in early apoptotic cells was observed in the bvPLA2-treated group.

### 2.5. Reduction of Activated Caspase-3 Expression by bvPLA2 Treatment

**Figure 5:**
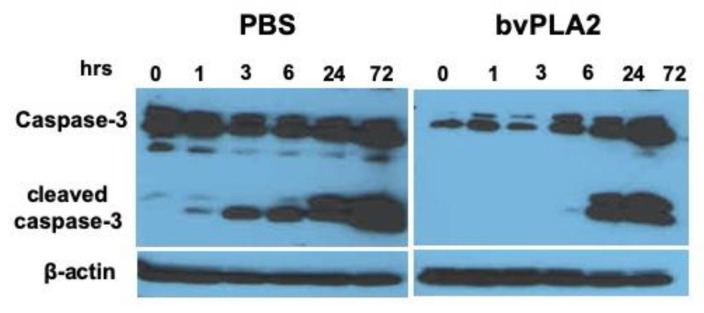
Expression of caspase-3 in mouse splenocytes in the absence or presence of bvPLA2. Mouse splenocytes were treated with PBS or bvPLA2 and incubated at 37 °C for 0, 1, 3, 6, 24, and 72 h and then analyzed by western blotting with a monoclonal anti-caspase-3 antibody. Typical immunoblot of each sample showing the 32-kDa procaspase band and 12 and 17 kDa cleaved-caspase-3 bands. β-actin was used as a loading control for normalization. Data are representative of two individual experiments.

We measured caspase-3 cleavage as an indicator of apoptosis induction since caspase-3 induction plays an important role in a number of upstream pathways of the final execution of apoptosis. Caspase-3 is synthesized as a 32-kDa proenzyme which is cleaved into 12 and 17 kDa subunits that are associated to form functionally active caspase-3 enzyme. Figure 5 shows the kinetics of caspase activation and caspase-3 proteolytic cleavage dramatically reduced by bvPLA2. The cleavage of caspase-3 was evident already at 1 h in the control-treated splenocytes. However, it was only detected 24 h after bvPLA2 treatment. Moreover, the expression of activated caspase-3 was significantly higher than that of procaspase-3 after 24 h.

### 2.6. bvPLA2 Induced the Expression of PD-1 and CTLA-4 in Splenocyte Culture

**Figure 6:**
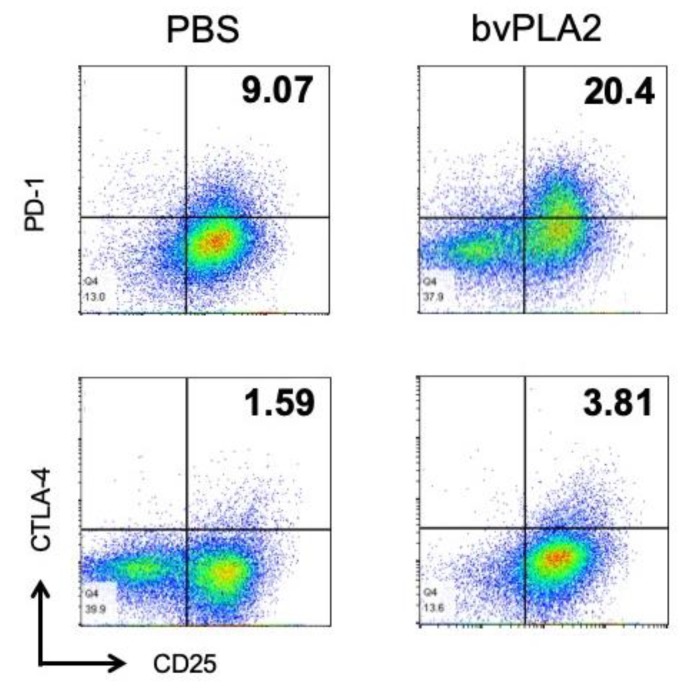
Effect of bvPLA2 treatment on the levels of CTLA-4 and PD-1 in vitro. Flow cytometry of mouse splenocytes that were stimulated with anti-CD3/CD28 antibodies in the presence of bvPLA2. Representative dot-plots showed staining of CD25 and CTLA-4 or PD-1 expression. Live cells were gated with anti-CD4 antibody and subsequent gating based on expression of CD25 and CTLA-4 or PD-1 is depicted.

Differential expression of the T cell-inhibitory receptors, PD-1 and CTLA-4, was determined by flow cytometry. Figure 6 shows that PD-1 and CTLA-4 expressions on CD4^+^CD25^+^ Treg cells were increased by bvPLA2 treatment.

### 2.7. Involvement of IL-2 in bvPLA2-Induced Suppression of Apoptosis

**Figure 7:**
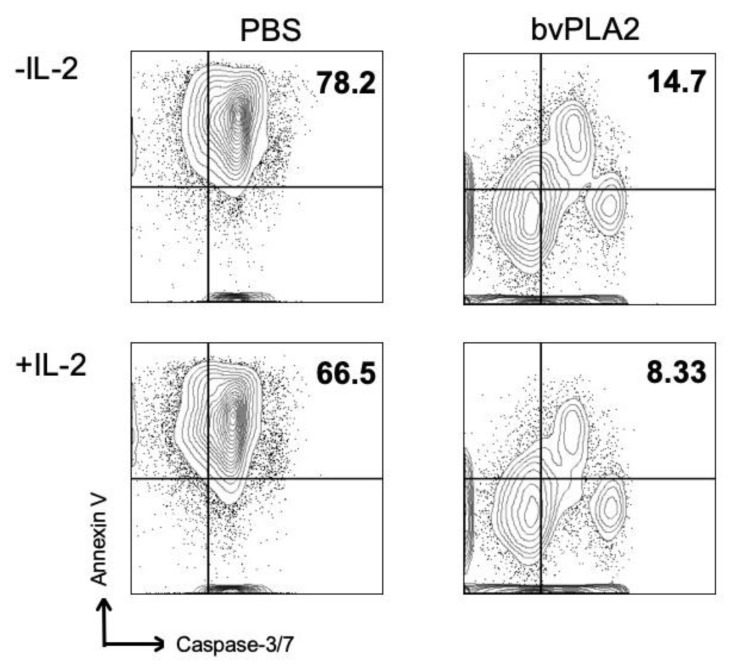
Involvement of the IL-2 signal in the reduction of apoptosis by bvPLA2 treatment in vitro. CD4^+^ T cells were isolated from mouse splenocytes by negative magnetic-activated cell sorting (MACS) sorting, then labeled with anti-CD25 magnetic beads and isolated. Isolated CD4^+^CD25^+^ cells were treated with bvPLA2 for 72 h in the presence of anti-CD3/CD28 stimulation, then stained with Annexin V, 7-AAD, and active-caspase-3 antibody. Live cells were gated at 7-AAD negative populations. The early apoptotic cells were depicted as Annexin V and active-caspase-3 double-positive populations.

CD25 is the high-affinity IL-2 receptor α-chain. IL-2-mediated signaling is critical in the generation process of effector and memory Tregs, and IL-2 plays an important role in the generation and maintenance of Treg cells. To assess the involvement of IL-2 in bvPLA2-mediated suppression of apoptotic signaling, highly purified magnetic-activated cell sorting (MACS)-sorted CD4^+^CD25^+^ Treg cells were stimulated with anti-CD3/CD28 antibodies in the presence or absence of recombinant mouse IL-2 (rmIL-2) for 3 days. Early apoptosis was measured using Annexin-V and caspase-3/7 antibody. Treatment with bvPLA2 reduced early apoptosis in CD4^+^CD25^+^ Treg cells (Figure 7). However, no significant differences were observed in both PBS and bvPLA2-treated Treg cells with or without rmIL-2 treatment.

## 3. Discussion

To elucidate the regulatory mechanisms of bvPLA2 in Treg induction, we examined the role of apoptotic signaling during bvPLA2 treatment using splenocyte cultures. The mTOR inhibitor rapamycin was used as positive control in this study since it promotes the in vitro expansion of Foxp3^+^ Treg cells in murine and human and maintain the suppressive capacity and their regulatory phenotype [27]. In vitro studies demonstrated that bvPLA2 treatment selectively increased Treg population and simultaneously decreased apoptotic cell death, as demonstrated by decrease in caspase-3 expression and Annexin V-positive early apoptotic populations. Further characterization of expanded Tregs showed that PD-1 and CTLA-4 were more broadly expressed in cells treated with bvPLA2. This result is in contrast with rapamycin which had been previously shown to have no significant effect on the expression of PD-1 or CTLA-4 [28,29]. Moreover, the bvPLA2-mediated reduction of apoptosis in Treg cells was independent of IL-2. Furthermore, the bvPLA2-treated Tregs survived for a longer period than the untreated Tregs in vivo. Taken together, these data suggest that bvPLA2 plays an important role in the regulation of apoptosis in expanded Tregs.

The major components of the bee venom (BV) include phospholipase A2 (PLA2), which is approximately 10–12% of the dry weight of the BV in the European honeybee, or *Apis mellifera* [30]. PLA2 derived from BV, called bvPLA2, belongs to Group III of secretory PLA2 (sPLA2). Group III of sPLA2 has been reported to be involved with a wide range of cellular responses including signal transduction, pain relief, host defense, and blood coagulation [31]. The therapeutic effect of bvPLA2 extends to the treatment for neurodegenerative diseases including AD [32]. Previous studies demonstrated the neuroprotective effect of bvPLA2 against Parkinson’s Disease [33] and AD [9]. The main action of bvPLA2 on a cellular level is the suppression of immune responses through the stimulation of dendritic cells, which ultimately leads to an increased function of Treg cells [20]. Previous reports showed that treatment with bvPLA2 induce the expansion of immunosuppressive Treg cells in vitro and in vivo [8]. In this study, the proliferation and the extended survival period of Treg populations by bvPLA2 treatment are novel findings in addition to the previous studies which stated the effect of bvPLA2 in various immune disease-related models [8,12].

Several subtypes of CD4^+^Foxp3^+^ Treg cells have been described based on phenotype and function. Tregs developed in the thymus are referred to as natural or thymus Tregs (nTregs or tTregs), and Tregs derived outside of the thymus are called peripheral or induced Treg cells (pTregs or iTregs). In addition to the development of Treg cells in the thymus, antigen stimulation of mature naïve T cells induces pTreg cell differentiation. Tissue-resident Treg cells have various phenotypes and functional capacities in comparison with conventional Treg cells. Specialized populations of Tregs have been identified in adipose tissue, skin, muscle, intestines, and central nervous system [34,35,36]. Additionally, CD4^+^Foxp3^−^ suppressive T cells have been identified, including Tr1, iTR35, and TH3 cells that secrete IL-10, IL-35, and TGF-β, respectively.

The role of Treg cells in the onset and progress of AD is not yet been completely elucidated. In a previous study, higher frequency of Treg and increased suppressive activity in neurodegeneration in the elderly patients have been reported [37]. Another study identified that PD-1 Tregs are found increased in both severe AD patients and mild cognitive impairment (MCI) patients when compared with healthy controls [38]. Recent studies have supporting evidence with the results of our study, showing that Treg cells play a beneficial role in the pathophysiology of AD by modulating the microglial response to Aβ deposition and slowing the progression of the disease [39]. Another study showed that systemic transplantation of autologous Tregs in a murine model upon human umbilical cord-derived mesenchymal stem cells education attenuated the cognitive deficit, Aβ deposition, and microglial activation in AD mouse models [40]. TGF-β1, secreted by Treg cells, showed anti-neuroinflammation activity in an AD model [41]. These findings emphasize the neuroprotective effect of Treg cells, and are in line with our study, which shows Tregs as the potentially applicable treatment to AD patients.

PD-1 is a co-inhibitory receptor of the CD28/B7 family that negatively regulates T cell activation through interaction with its two known ligands, PD-L1 (CD274) and PD-L2 (CD273). PD-1 expression can be induced by T-cell receptor (TCR) stimulation and by the common γ chain cytokines (IL-2, IL-7, IL-15, and IL-21) [42]. Moreover, PD-1 is expressed on Tregs and inhibits their function in low-dose IL-2 therapy. Specifically, PD-1 regulates the proliferation and apoptosis of IL-2-induced Treg cells in murine models and in patients receiving low-dose IL-2 therapy. These findings suggest that PD-1 signaling has a critical role in the maintenance of Treg homeostasis and immune tolerance. Furthermore, PD-1 blockade completely abolishes the effects of IL-2, promotes Treg apoptosis, and reduces the number of Treg cells in vivo. However, the mechanisms by which Tregs modulate the expression of PD-1 during exogenous administration of IL-2 remain to be clarified. CTLA-4 is a member of the immunoglobulin superfamily and is a type 1 transmembrane glycoprotein that interacts with CD80 and CD86. Activated naïve T cells upregulate CTLA-4, especially upon TCR engagement, whereas nTreg cells constitutively express high levels of CTLA-4. High levels of CTLA-4 on Treg cells suggest that CTLA-4 may play a critical role in Treg-mediated suppressive function. The disruption of CTLA-4 specifically on Tregs leads to the spontaneous development of systemic lymphoproliferation, hyperproduction of IgE, and fatal cell-mediated autoimmune disease [43]. In this study, bvPLA2 treatment also induced the expression of PD-1 and CTLA-4. The relationship between induction of PD-1 and CTLA-4 on Treg cells and apoptotic signaling pathway needs to be elucidated in the future.

Activated lymphocytes often express increased levels of death receptors, rendering them susceptible to apoptosis [44,45]. Apoptosis is a highly conserved form of programmed cell death and a major regulator of Treg cell homeostasis. Furthermore, IL-2-mediated induction of the anti-apoptotic Bcl-2 protein Mcl-1 is crucial for Treg cell survival. In this study, we evaluated the role of the apoptosis pathway in bvPLA2-induced Treg expansion. Tregs are highly sensitive to early apoptosis, and this study shows that bvPLA2 treatment reduced the early apoptosis on CD4^+^CD25^+^ Treg cells and increased the expression levels of PD-1 and CTLA-4, suggesting a pivotal role of these molecules in bvPLA2-mediated Treg expansion. Translating the knowledge about immune tolerance obtained from basic research to clinical application promises safer and more effective therapies in medicine. The results of this study suggest that the administration of mouse Treg cells may be potentially applicable to the treatment of immune system-related diseases including Alzheimer’s disease.

## 4. Materials and Methods

### 4.1. Animals

Male Foxp3^EGFP^ C57BL/6 mice (~6–7 weeks of age) were purchased from The Jackson Laboratory (Bar Harbor, ME, USA). Mice were maintained under pathogen-free conditions with air conditioning and 12-h light/dark cycle. All mice had free access to food and water during the experiments. All procedures were performed in accordance with the Rules for Animal Care and Guiding Principles for Animal Experiment Using Animals and were approved by the University of Kyung Hee Animal Care and Use Committee (KHASP(SE)-17-149), approval date: 15 November 2017. 

### 4.2. Preparation of bvPLA2

Phospholipase A2, from honey bee venom (*Apis mellifera*) was purchased from Sigma (St. Louis, MO, USA). The preparation process of the bvPLA2 is as follows [33,46]. The first step is isolation and purification, which involves dissolving raw BV in high performance liquid chromatography (HPLC) grade water at a concentration of 1 mg/mL; the second step is filtration, during which the diluted samples are applied to PTFE membrane filter (pore size 0.45 μm); the next step is concentration, during which the filtered mixtures are concentrated by a tangential flow filtration (TFF) system to reduce the volume. For sterile filtration, the mixtures are fitted with ultrafiltration membrane. The purified bvPLA2 is freeze-dried and collected as a white powder. The content of bvPLA2 is determined using the HPLC system and diluted to a concentration of 0.1 mg/mL. Undesired substances are removed by membrane filters (pore size 0.22 μm PVDF sterile membrane filter). The separation and detection are carried out on reversed-phase HPLC system using a liquid chromatograph and a UV-visible detector. The sample is chromatographed at 25 °C at a flow rate of 2 mL/min. The elution is performed with a linear gradient of 0%–80% acetonitrile in 0.1% trifluoroacetic acid, and the elution profile is monitored at 220 nm. The area of the peak is used to measure the recovery of bvPLA2. The physical state of the final content of bvPLA2 was white powder and it was stored in freezing condition below −20 °C. It consisted of 93% of PLA2 and 7% of minor unknown constituents, as shown in previous report [33].

### 4.3. Cell Cultures

Mice were anesthetized with isoflurane and sacrificed. The spleens were removed and disrupted using a 40-μm cell strainer (Corning, NY, USA). After lysing the red blood cells, the splenocytes were washed with phosphate buffered saline (PBS) and resuspended in RPMI-1640 (WelGENE INC., Taegu, Korea) supplemented with 10% fetal bovine serum (FBS), 50 IU/mL penicillin, and 50 μg/mL streptomycin (Hyclone, Logan, UT, USA). Splenocytes were treated with 0.4 μg/mL bvPLA2 (Sigma-Aldrich, ST. Louis, MO, USA) in the presence of plate-bound CD3 (5 μg/mL) and soluble CD28 (2 μg/mL) mAbs (both from BD Biosciences, San Jose, CA, USA). Cells were stimulated with 100 nM rapamycin alone as positive control. Splenocytes were incubated at 37 °C for 72 h and cells were harvested for the next experiments.

### 4.4. Adoptive Transfer of Tregs

Splenocytes from Foxp3^EGFP^C57BL/6 mice were cultured with bvPLA2 or PBS for 72 h as described above. Then, FoxP3-EGFP^+^ Treg cells were sorted by FACSAriaⅡ (BD Biosciences, San Jose, CA, USA); 1 × 10^6^ Tregs were adoptively transferred to C57BL/6 mice via the tail vein. After 7, 28, and 42 days, the mice were sacrificed and splenocytes were isolated and analyzed by flow cytometry.

### 4.5. Flow Cytometry

Single-cell suspensions from spleens were aliquoted into tubes and washed once in stain buffer (BD Biosciences). Cells were incubated with fluorescence-labeled antibodies at 4 °C for 30 min. Antibodies used for flow cytometry included: anti-CD4-PE-Cy7, anti-CD25-APC-Cy7, anti-CD127-PE, anti-CD62L-APC, anti-CTLA-4-PE, and anti-PD-1-PE. To trace of adoptively transferred Treg, single-cell suspension from spleens were labeled with anti-CD4-APC. Apoptosis was determined by labeling with Annexin V-APC, PI, and anti-activated caspase-3/7-FITC antibody (BD Biosciences, San Jose, CA, USA). Appropriate isotype control antibodies were used to define marker settings. Stained cells were sorted by BD FACS Calibur flow cytometer (BD Biosciences) and the data were analyzed using FLOWJO software (v10, Tree star, Ashland, OR, USA).

### 4.6. Western Blotting

Cells were lysed in Pro-PREP Protein Extraction Solution (iNtRON Biotechnology, Seoul, Korea) and kept at 4 °C for 30 min. Protein concentration was measured by the Bradford method, using bovine serum albumin (BSA) as a standard. Equal amounts of extracted proteins were separated by 15% sodium dodecyl sulfate polyacrylamide gel electrophoresis (SDS-PAGE) and transferred onto a nitrocellulose membrane (Bio-Rad, Hercules, CA, USA) using a tank blot procedure (Bio-Rad Mimi Protean). Then, the membranes were blocked with 3% non-fat milk in PBS containing 0.05% Tween-20 for 1 h at room temperature (RT). Membranes were incubated with antisera against caspase-3 (Cell Signaling, Boston, MA, USA) and β-actin (Santa Cruz Biotechnology, Santa Cruz, CA, USA) at 1:1000 dilution for 2 h at RT. The washed membranes were incubated with 1:5000 dilution of respective horseradish peroxidase-linked secondary antibodies for 1 h. The specific bands were visualized by enhanced chemiluminescence reagents (ECL, Amersham Pharmacia Biotech, Piscataway, NJ, USA). The same membrane was re-probed with a β-actin-specific antibody as a loading control.

### 4.7. CD4^+^CD25^+^ Treg Cell Preparation

CD4^+^CD25^+^ Treg cells were isolated from the spleens obtained from male Foxp3^EGFP^ C57BL/6 mice using magnetic-activated cell sorting (MACS) according to manufacturer’s protocols (CD4^+^CD25^+^ Regulatory T Cell Isolation Kit; Miltenyi Biotec, USA). Briefly, CD4^+^ T cells were negatively selected using a biotinylated antibody cocktail and anti-biotin microbeads and then separated into CD4^+^CD25^+^ T cells. CD4^+^CD25^+^ T cells were positively selected using PE-labeled anti-CD25 antibody and anti-PE microbeads. The purity of Treg populations was >78%, as determined by flow cytometry. To investigate the involvement of IL-2 in bvPLA2-mediated suppression of apoptosis, isolated CD4^+^CD25^+^ cells were treated with bvPLA2 and/or 1000 U of rmIL-2 (R&D Systems, Minneapolis, MN, USA) in the presence of anti-CD3/CD28 stimulation.

### 4.8. Statistical Analysis

Data derived from two or more than two groups with normal distributions were compared with one-way ANOVA followed by Tukey’s Multiple Comparison tests and Student’s *t*-test using the Prism 5.01 software (GraphPad Software Inc., San Diego, CA, USA). Differences with a *p*-value of ≤ 0.05 were considered statistically significant. Data were expressed as the arithmetic means of triplicates ± SEM.
